# Genome analysis of the metabolically versatile *Pseudomonas umsongensis* GO16: the genetic basis for PET monomer upcycling into polyhydroxyalkanoates

**DOI:** 10.1111/1751-7915.13712

**Published:** 2021-01-06

**Authors:** Tanja Narancic, Manuel Salvador, Graham M. Hughes, Niall Beagan, Umar Abdulmutalib, Shane T. Kenny, Huihai Wu, Marta Saccomanno, Jounghyun Um, Kevin E. O'Connor, José I. Jiménez

**Affiliations:** ^1^ BiOrbic – Bioeconomy Research Centre University College Dublin Belfield Dublin 4 Ireland; ^2^ UCD Earth Institute and School of Biomolecular and Biomedical Science University College Dublin Belfield Dublin 4 Ireland; ^3^ Faculty of Health and Medical Sciences University of Surrey Guildford GU2 7XH UK; ^4^ UCD Earth Institute and School of Biology and Environmental Science University College Dublin Belfield Dublin 4 Ireland; ^5^ Bioplastech Ltd. NovaUCD, Belfield Innovation Park University College Dublin Belfield Dublin 4 Ireland; ^6^ Department of Life Sciences Imperial College London London SW7 2AZ UK

## Abstract

The throwaway culture related to the single‐use materials such as polyethylene terephthalate (PET) has created a major environmental concern. Recycling of PET waste into biodegradable plastic polyhydroxyalkanoate (PHA) creates an opportunity to improve resource efficiency and contribute to a circular economy. We sequenced the genome of *Pseudomonas umsongensis* GO16 previously shown to convert PET‐derived terephthalic acid (TA) into PHA and performed an in‐depth genome analysis. GO16 can degrade a range of aromatic substrates in addition to TA, due to the presence of a catabolic plasmid pENK22. The genetic complement required for the degradation of TA *via* protocatechuate was identified and its functionality was confirmed by transferring the *tph* operon into *Pseudomonas putida* KT2440, which is unable to utilize TA naturally. We also identified the genes involved in ethylene glycol (EG) metabolism, the second PET monomer, and validated the capacity of GO16 to use EG as a sole source of carbon and energy. Moreover, GO16 possesses genes for the synthesis of both medium and short chain length PHA and we have demonstrated the capacity of the strain to convert mixed TA and EG into PHA. The metabolic versatility of GO16 highlights the potential of this organism for biotransformations using PET waste as a feedstock.

## Graphical abstract

In this article we have summarised our findings resulting from analysing the complete genome sequence of *Pseudomonas umsongensis* GO16 and we have determined experimentally its potential for the upcycling of monomers resulting from PET hydrolysis. Besides a general genomic characterisation, we have identified and validated the set of genes required for terephthalate and ethylene glycol degradation as well as the ability of the strain for producing short and medium‐chain length polyhydroxyalkanoates.

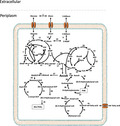

## Introduction

Polyethylene terephthalate (PET) is one of the most commonly used plastic polymers with an annual demand of approximately 33 million tonnes (Geyer *et al*., 2017). Even though PET bottles are used as an example of successful recycling, globally only 7% of PET produced annually is actually recycled (Forum, 2015) with a large majority of plastic waste still landfilled (Kasper, 2013; B, ; Plastics Europe, 2016). Among the options to reduce PET waste, the possibility of using microorganisms to both degrade and upcycle PET has gained attention (Narancic and O'Connor, 2017; Wei and Zimmermann, 2017; Blank *et al*., 2019). However, the ability to degrade PET is rare in nature (Wierckx *et al*., 2018; Salvador *et al*., 2019). PET belongs to the group of hydrolysable polymers and there are several examples of bacterial hydrolytic enzymes that were shown to break down PET into oligomers and monomers of terephthalate (TA) and ethylene glycol (EG; (Wei and Zimmermann, 2017). While the emergence of ‘plastic eating’ bacteria such as *Ideonella sakaiensis* (Yoshida *et al*., 2016) grabbed the attention of scientific and general audiences, there is still uncertainty about the rate of bacterial depolymerization of PET and the efficiency of PET monomer catabolism (Yang *et al*., 2016). Furthermore, the complete mineralization of PET to CO_2_ will not encourage circularity (Wierckx *et al*., 2015). Recycling of PET waste into a material such as a biodegradable plastic like polyhydroxyalkanoate (PHA) creates an opportunity to improve resource efficiency by extending and diversifying the life of the material, thus contributing to a circular economy (European Commission, 2015; Wierckx *et al*., 2015). It is necessary to optimize the enzymatic hydrolysis of PET but also to develop efficient microbial transformation of PET‐derived monomers TA and EG, arising from enzymatic degradation, into other molecules of value.

The strain *Pseudomonas umsongensis* GO16 was isolated from soil exposed to PET granules at a PET bottle processing plant (Kenny *et al*., 2008). The biotechnological potential of this strain was demonstrated by developing a process for the conversion of TA obtained from pyrolysis of PET into a biodegradable polymer, namely medium chain length polyhydroxyalkanoate (mcl‐PHA) (Kenny *et al*., 2008, 2012). However, the metabolic basis of TA conversion into PHA was not investigated.

Since the genus *Pseudomonas* was first described in 1894, over 190 species have been identified to date (Peix *et al*., 2018). The ability of pseudomonads to thrive in soil, sediments, hot springs, extremely cold environments, air, plants, animals and others, is largely due to their tremendous metabolic versatility allowing them to cope with harsh and stressful environmental conditions (Poblete‐Castro *et al*., 2017; Peix *et al*., 2018). The adaptability of the species belonging to the *Pseudomonas* genus to very different lifestyles has inspired the biotechnological use of these organisms as microbial cell factories for the production of chemicals, polymers, as bio‐controlling agents, as well as in bioremediation (Poblete‐Castro *et al*., 2017).

In this study, we have conducted a genome analysis of *P. umsongensis* GO16 and identified not only the genes responsible for TA and EG metabolism but also for catabolism of a wide range of aromatics. Moreover, we have identified the set of genes responsible for the synthesis of both short chain length (scl) and mcl‐PHA, and experimentally validated the accumulation of these biopolymers from different substrates including an equimolar mixture of TA and EG.

## Results and discussion

### Overall genomic organization

The *P. umsongensis* GO16 genome characteristics are given in Table 1. The genome shows a very high similarity with the previously reported *P. umsongensis* DSM 16611, shown to degrade a wide range of xenobiotics, such as phenol, trinitrotoluene, xylene, polyaromatic hydrocarbons and petroleum (Furmanczyk *et al*., 2017). The GC content of these two strains is similar, however *P. umsongensis* GO16 genome is 650 485 bp longer (Table 1). The strain GO16 contains a 7.3 Mbp chromosome (GenBank: CP044409.1) and an 82 kbp plasmid named pENK22 (GenBank: CP044408.1).

**Table 1:** *P. umsongensis* GO16 genome features and comparison with the genome of *P. umsongensis* DSM 16611.

Features	*P. umsongensis* GO16	*P. umsongensis* DSM 16611
Length (bp)	7 269 974 chromosome + 81 914 pENK22	6 701 403
GC content (%)	59.2	59.7
CDS	6867	6152
rRNA genes	6	7
tRNA genes	57	62

**Fig. 1:**
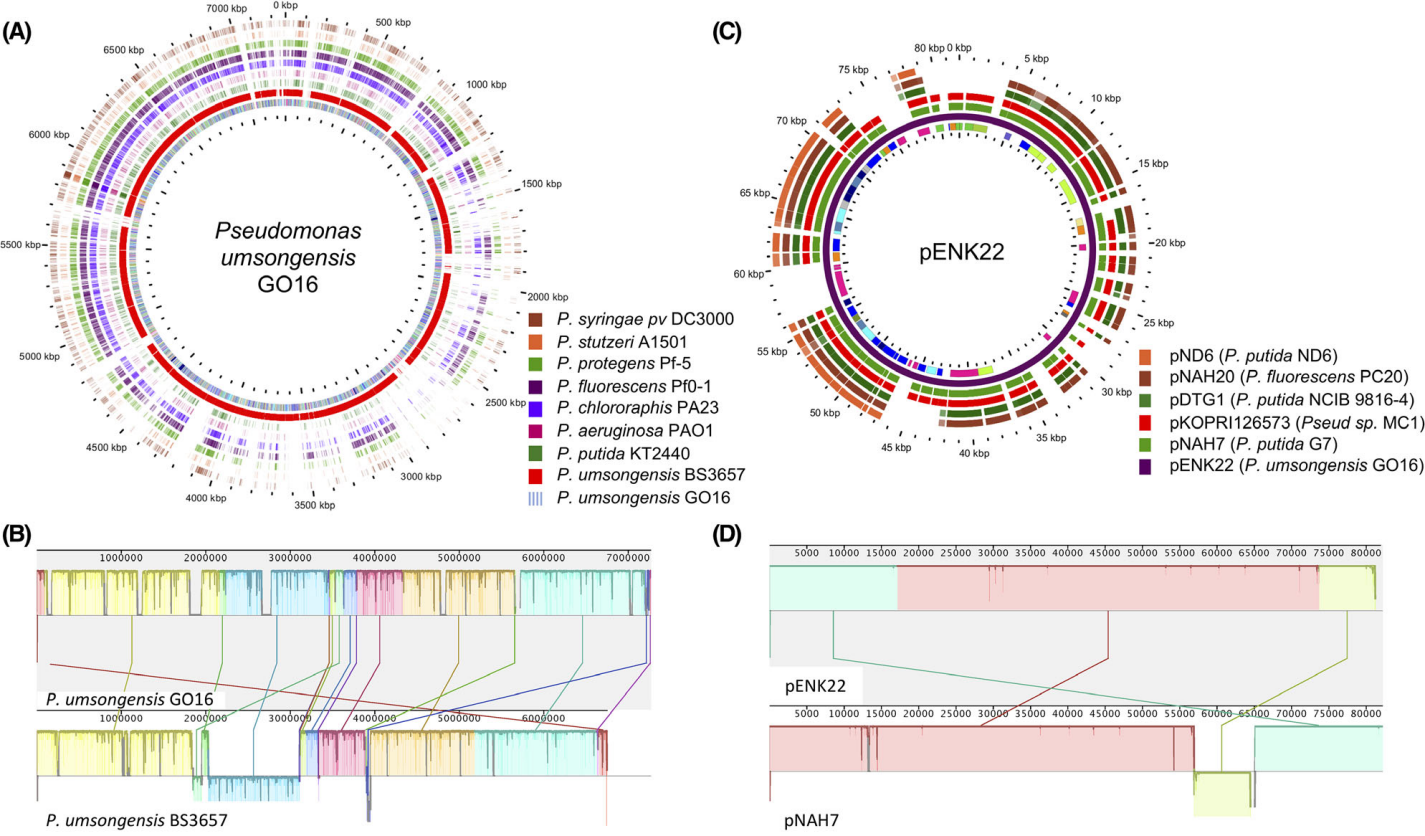
Global analysis of the *P. umsongensis* GO16 genome. A. BLAST atlas comparing genes present in strain GO16 (used as a reference) with other representative *Pseudomonas* strains. The circles represent the genomes of the species shown in the insert starting with *Pseudomonas syringae* pv tomato DC3000 (outer circle) and finishing with *P. umsongensis* GO16 shown as the set of clusters of orthologous groups (COGs; inner circle). B. Genome alignment of strains of *P. umsongensis* GO16 (upper bar) and BS3657 (lower bar). C. BLAST atlas comparing present genes in plasmid pENK22 with other representative plasmids of *Pseudomonas* species containing naphthalene‐catabolic genes (*nah*). D. Detailed alignment between pENK22 and the naphthalene degrading plasmid NAH7. Genes, COG (represented by the innermost circle) and blast analysis resulting from the analysis are summarized in Tables S1 and S2.

The genome sequence was compared to notable organisms belonging to the same genus and for which a closed genome sequence is available (Fig. 1A). *P. umsongensis* GO16 possesses the largest genome of all of them, even larger than that of *P. protegens* Pf‐5 (7 074 893 bp). The phylogenetic analysis conducted with coding sequences of 30 species of the *Pseudomonas* genus places GO16 as a member of the *P. umsongensis* species, closely related to *P. mandellii* and *P. frederiksbergensis* (Fig. S1). Even though there is a high degree of functional conservation across species (Table S1), *P. umsongensis* GO16 has a distinct set of genome sections when compared *P. umsongensis* BS3657, which is the closest species for which a complete genome is available (Fig. 1B). The main differences, in addition to rearrangements, correspond to the insertion of segments likely resulting from horizontal gene transfer events in the chromosome of GO16. This is the case of a prophage (located in coordinates 805 892–830 810 bp); IS6 and IS2 transposons (1 212 038–1 253 316 bp), a region containing conjugative integrative elements, group II introns, IS3, IS5 and IS110 transposons (1 815 827–2 062 259 bp), IS3 transposon (2 661 452–2 775 901 bp); a region containing IS1182, IS110 transposons and an integrative element (4 806 223–4 889 799 bp); and regions with integrative elements (5 642 431–5 745 063 and 7 046 312–7 057 127 bp).

The plasmid pENK22 of *P. umsongensis* GO16 has a size of 81 914 kb and shows 99% identity with the 81 kb plasmid from *Pseudomonas* sp. MC1 (Ahn *et al*., 2017) and the 82 kb *P. putida* G7 NAH7 plasmid (Sota *et al*., 2006) (Fig. 1C). Like those plasmids, pENK22 encodes for a complete pathway for naphthalene mineralization and has a genetic organization almost identical to NAH7 (Sota *et al*., 2006) with the exception of a gene rearrangement (Fig. 1D; Table S2).

### Central metabolism

**Fig. 2:**
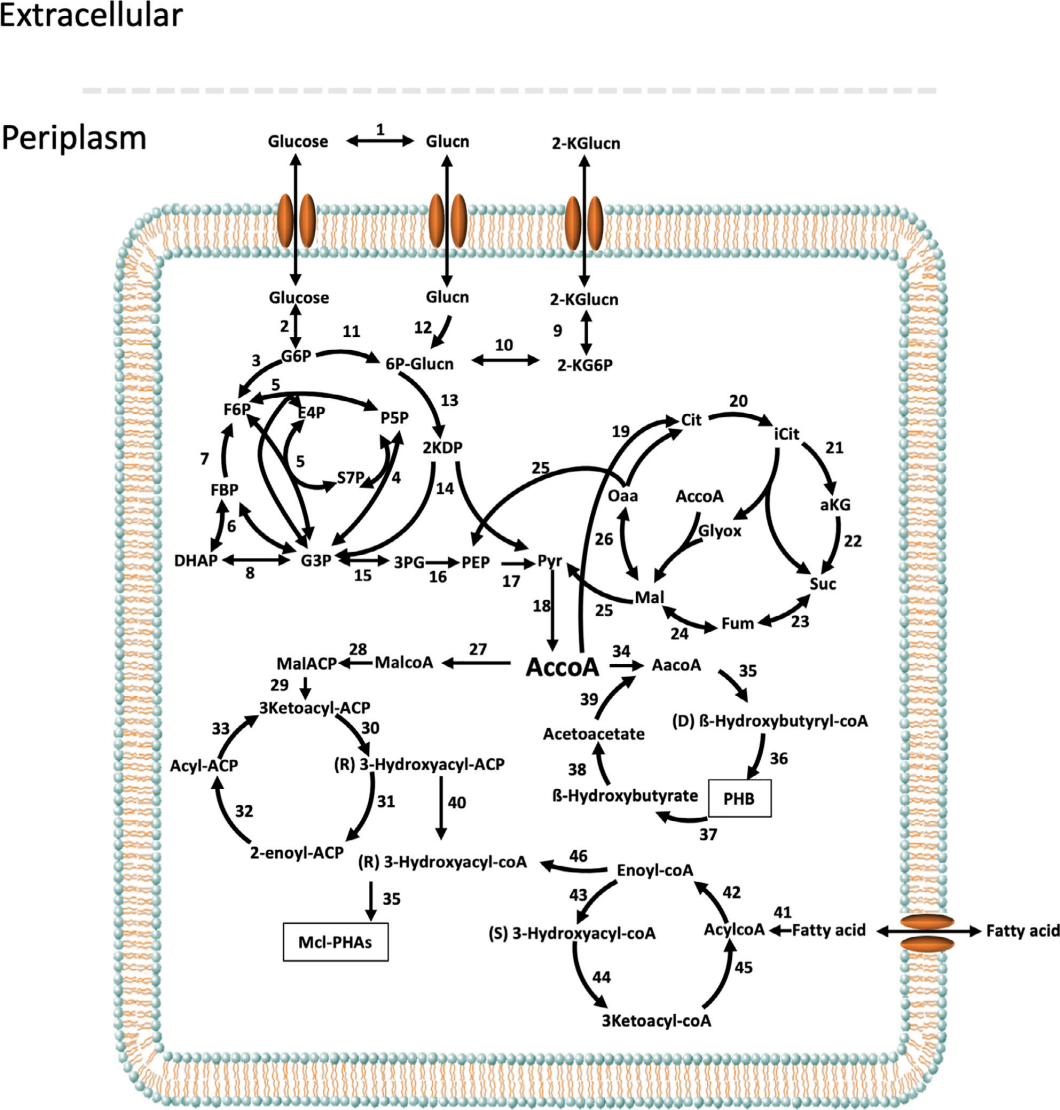
Proposed central metabolism of *P. umsogensis* GO16. The complete list of the enzymes and isozymes catalyzing each reaction is provided in Table S3. Abbreviations are as follows: Glucn, gluconate; 2‐KGlucn, 2‐keto‐gluconate; 2‐KG6P, 2‐keto‐gluconate‐6P; G6P, glucose‐6‐P; F6P, fructose‐6‐P; FBP, fructose‐1,6‐P2; DHAP, dihydroxyacetone‐P; 6P‐Glucn, 6‐phosphogluconate; 2KDP, 2‐keto‐3‐deoxy‐6‐phosphogluconate; P5P, Pentose‐5‐P; S7P, sedoheptulose‐7‐P; E4P, erythrose‐4‐P; G3P, glyceraldehyde‐3‐P; 3PG, glycerate‐3‐P; PEP, phosphoenolpyruvate; AccoA, acetyl‐coenzyme A; Aacoa, acetoacetyl‐coA; Oaa, oxaloacetate; aKG, α‐ketoglutarate; iCit, Isocitrate; Cit, Citrate; Glyox, Glyoxilate; Suc, Succinate; Fum, Fumarate; Mal, Malate; MalcoA, Malonyl‐coA; MalACP, Malonyl‐ACP; mcl‐PHAs, medium chain length Polyhydroxyalkanoate; PHB, Polyhydroxybutyrate..

The central metabolism of *P. umsongensis* GO16 was reconstructed based on the annotation derived from the genome sequence (Fig. 2; Table S3). Like in most *Pseudomonas* species, glucose is likely metabolized by the Entner‐Doudoroff pathway (ED), the Embden‐Meyerhof‐Parnas (EMP) pathway and the pentose phosphate pathway (PP), which produce a surplus of reducing power that allows for coping with stressful environments (Nikel *et al*., 2015). In this metabolism glucose is mainly transformed to gluconate in the periplasm prior to its conversion in 6‐phosphogluconate. Similar to *P. putida* KT2440, GO16 lacks a cytoplasmic glucose dehydrogenase which could carry out that conversion inside the cell, and a phosphofructokinase that could transform fructose‐6‐phosphate into fructose‐1,6‐bisphosphate following the conventional EMP glycolytic pathway.

According to the functional annotation, the genome of GO16 has a large number of potentially redundant enzymes related to a likely diverse metabolism of lipids. There are a number of activities that could constitute a β‐oxidation pathway including, for example, 23 genes annotated as 3‐oxoacyl‐(acyl‐carrier protein) reductases, 5 genes coding for enoyl‐(acyl‐carrier protein) reductase III, 4 genes coding for 3‐oxoacyl‐(acyl‐carrier‐protein) synthase II, 17 genes for the β‐oxidation enzyme acetyl‐CoA C‐acetyltransferase and 10 for 3‐hydroxyacyl‐CoA dehydrogenases.

### Degradation of aromatics

**Fig. 3:**
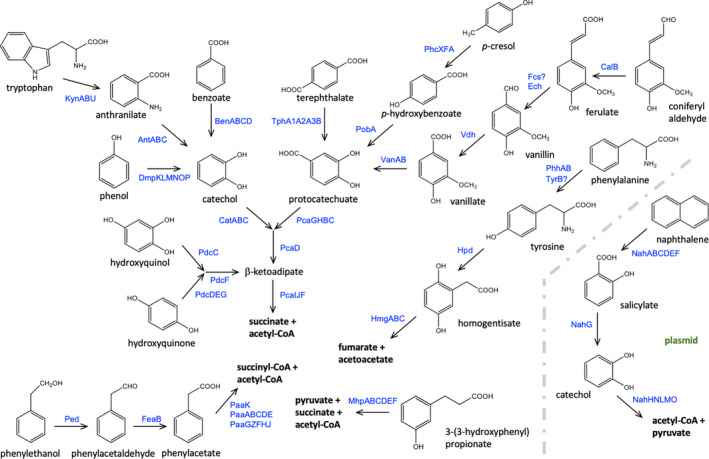
Proposed metabolism of aromatics in *P. umsongensis* GO16. The diagram shows the range of aromatic substrates metabolized by the bacterium. In blue are shown the required for the conversion of the substrates into central metabolites. The location and homology of the genes encoding for the corresponding enzymes as well as potential transcriptional regulators and transporters is shown in Table S4.

Given that *P. umsongensis* GO16 was isolated using the aromatic compound TA as the sole carbon and energy source, we analysed the genome for the presence of pathways involved in the mineralization of other aromatics. This species shows remarkable metabolic versatility comparable or even larger than other members of the genus, possibly due to the above average size of the genome (Fig. 3) (Jiménez *et al*., 2004).

Our analysis identified a set of central pathways likely responsible for the degradation of monoaromatic molecules (Fig. 3 and Table S4). These include the *ortho*‐ pathway for degradation of catechol (*cat*), the 3,4‐dioxygenolytic pathway for degradation of protocatechuate (*pca*) and a pathway for the degradation of hydroxyquinol and hydroquinone (*pdc*), all of which converge in the β‐ketoadipate pathway that leads to the central metabolism. In addition, we found putative central pathways for the metabolism of homogentisate (*hmg*), phenylacetate (*paa*), 3‐hydroxyphenylpropionate (*mhp*) and the *meta*‐ cleavage for catechol (*xyl*) contained in the plasmid pENK22. Notably, the likely *pcaGH* and *pcaIJ* genes encoding, respectively, for the protocatechuate 3,4‐dioxygenase and 3‐oxoadipate CoA‐transferase involved in protocatechuate degradation were duplicated in the genome (Table S4).

These pathways are used for funnelling a plethora of aromatic molecules towards central metabolism. We identified genes potentially involved in the degradation of benzoate, tryptophan, anthranilate and phenol leading to catechol; ferulate, vanillin, vanillate, coniferyl aldehyde, *p*‐hydroxybenzoate (pOHB) and *p*‐cresol in addition to terephthalate degraded *via* protocatechuate; phenylalanine and tyrosine metabolized *via* homogentisate; phenylethanol and phenylacetaldehyde mineralized through phenylacetate. Naphthalene and salicylate are metabolized through the putative *meta*‐pathway for catechol encoded by the plasmid (Fig. 3 and Table S4). We confirmed experimentally that, in addition to TA, the GO16 strain can grow in 24 h on benzoate, tryptophan, pOHB, vanillate, protocatechuate, phenylacetate, phenylalanine and tyrosine (Fig. S2A and B). The strain exhibited low but detectable growth on the lignin derivatives *p*‐coumarate and ferulate (Fig. S2C). Likewise, we confirmed growth when in the presence of naphthalene vapours over a period of 4 days (Fig. S2D). The strain was unable to use vanillin, 2‐phenylethanol, anthranilate, *p*‐cresol, 3‐phenylpropionate and coniferyl aldehyde as sole carbon source in the conditions tested.

The pENK22 plasmid contains two putative operons for the catabolism of naphthalene, the upper pathway encoded by genes *nahA_a_* – *nahD* for the conversion of naphthalene to salicylate, and the lower pathway encoded by genes *nahG* – *nahY* for the conversion of salicylate to pyruvate and acetaldehyde *via* a *meta*‐cleavage pathway spanning over 26.4 kb (Fig. 3; Table S4). This pathway organization was identified in several bacterial strains capable of aerobic degradation of aromatic compounds (Williams and Sayers, 1994; Sota *et al*., 2006).

### TA and EG metabolism

As already mentioned, TA and EG are PET constituent monomers and can be obtained by pyrolysis or enzymatic hydrolysis of PET (Kenny *et al*., 2008; Kenny *et al*., 2012; Wei and Zimmermann, 2017). Genome mining of GO16 revealed the presence of genes for the complete mineralization of both, and the capacity of GO16 to use EG as a sole source of carbon and energy was also validated experimentally.

**Fig. 4:**
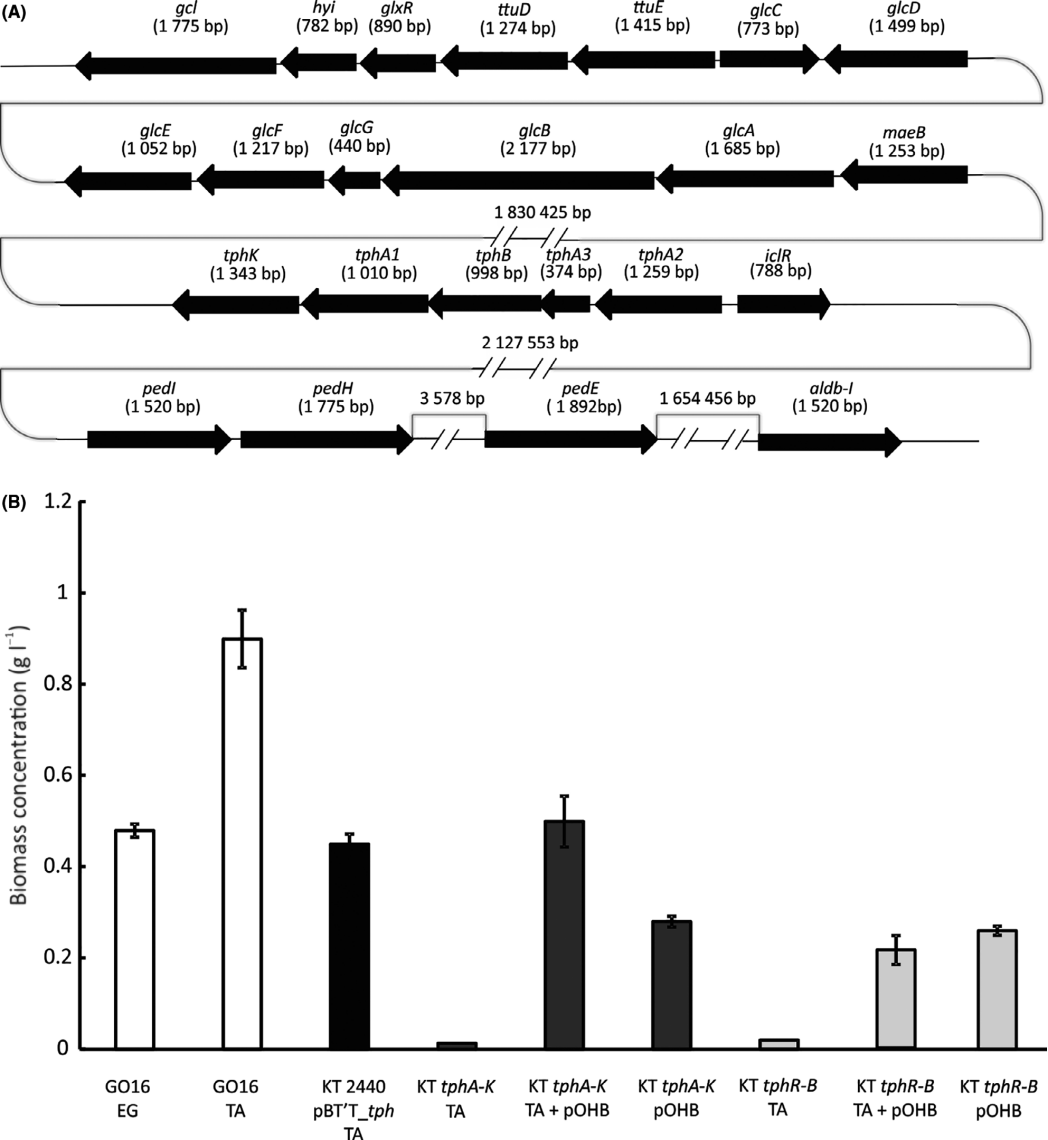
(A) Organization of genes in *P. umsongensis* GO16 that encode enzymes that enable growth with EG and TA when used as a sole source of carbon and energy. (B) Biomass (CDW g l^−1^) when *P. umsongensis* GO16 was grown with EG, TA and growth of *P. putida* KT2440 pBT’T_*tph*, KT2440 expressing whole *tph* operon including the native regulator from *P. umsongensis* GO16 grown with TA. *tphA‐K* represents KT2440 expressing the *tph* operon without the native regulator and this strain was grown with TA, *p*‐hydroxybenzoate (pOHB) or a mixture of both substrates. *tphR‐B* represents the growth of KT2440 expressing the *tph* *operon* without the transporter *tphK* *grown with TA,* pOHB or a mixture of both. The values represent the average of three independent biological replicates and correspond to the biomass accumulated in 48 h.

The operon for the catabolism of TA to protocatechuate (PCA) is 6085 bp long (Fig. 4A; Table S4). The catabolic genes are preceded by a regulator that belongs to the isocitrate lyase regulator‐type (IclR‐type) transcriptional regulators, in general involved in regulation of carbon metabolism, multidrug resistance, quorum sensing, etc. (Molina‐Henares *et al*., 2006). In *Pseudomonas* strains IclR‐type regulators are frequently involved in the regulation of the β‐ketoadipate pathway (Molina‐Henares *et al*., 2006). The regulator is followed by terephthalate 1,2‐dioxygenase subunits α (*tphA2*) and β (*tphA3*), a reductase component (*tphA1*), a dehydrogenase (*tphB*) and terephthalate transporter (*tphK*). Similar organization of TA degradation operon was reported for *Comamonas testosteroni* strains YZW‐D and E6, and *Rhodococcus opacus*, where TA is degraded to 1,2‐dihydroxy‐3,5‐cyclohexadiene‐1,4‐dicarboxylic acid by the action of terephthalate dioxygenase (TphA2A3A1), followed by the activity of TphB to convert this intermediate into protocatechuate (Sasoh *et al*., 2006).

While a permease *tphC* is involved in the facilitated diffusion of TA in *C. testosteroni* strains, in GO16 the transport of TA is mediated by an MFS transporter of the aromatic acid:H^+^ symporter (AAHS) family (*tphK* in Table S4), which shows homology to the *p*‐hydroxybenzoate transporter *pcaK* (Salvador *et al*., 2019) (Fig. 4A; Table S4).

We selected *P. putida* KT2440 for the recombinant expression of the *tph* genes identified in GO16. KT2440 has been certified as a safe microorganism and is considered a workhorse for biotransformations (Belda *et al*., 2016; Volke *et al*., 2020; Weimer *et al*., 2020). While *P. putida* KT2440 is equipped with numerous dioxygenases and can grow with a range of aromatic hydrocarbons, it cannot utilize TA. In fact, out of the four *tph* catabolic genes, only *tphA2* and *tphA1* have homologs in the chromosome of KT2440. They show identities of, respectively, 35% and 26% with the subunits BenA and BenC of the benzoate 1,2‐dioxygenase. When the whole *tph* operon, including the transporter and the regulator, was expressed in *P. putida* KT2440 on plasmid pBT'T_*tph* it conferred this organism the ability to use TA as a sole source of carbon and energy (Fig. 4B). The growth rate of the KT2440 pBT’T_*tph* with TA as a sole source of carbon and energy was 0.3 h^−1^, which is 1.7‐fold lower compared to glucose as a sole source of carbon and energy (Fig. S3). The final biomass reached by KT2440 pBT’T_*tph* was 1 g l^−1^, which is 1.6‐fold lower than the final biomass of GO16 when TA is used as a sole source of carbon and energy (Fig. S3, Table 3). It is worth noting that the whole *tph* operon, including the transporter *tphK* and the native regulator *iclR* (Fig. 4A) were required to allow growth of KT2440 pBT’T_*tph* with TA as the carbon source (Fig. 4B). A construct lacking the transporter *tphK* (fragment *tphRA2A3BA1*) failed to grow on TA as the sole carbon source. Moreover, no growth was observed in TA when the catabolic and transport *tphA2A3BK* genes cloned into pBT’T were expressed constitutively from the strong promoter *P_tac_*. However, this construct showed increased biomass formation (0.5 g l^−1^ CDW) when co‐cultured in TA (4.2 g l^−1^ corresponding to 1.96 g_C_ l^−1^) and pOHB (0.7 g l^−1^, providing 0.42 g_C_ l^−1^) used as an inducer of the *pca* genes, compared to the culture with pOHB alone (0.28 g l^−1^ CDW; Fig. 4B) (Kim *et al*., 2006). These results suggest the *tphR* can coordinate the expression of the *tph* genes and the downstream *pca* pathway required for PCA degradation in *P. putida* KT2440. Although a specific regulator for the expression of *pcaGH* in this organism has not been identified (Jimenez *et al*., 2002), TphR shares a 53% similarity with PcaU, another regulator of the IclR‐family known to control the expression of *pcaGH* in *Acinetobacter* sp. ADP1 in response to PCA (Gerischer *et al*., 1998). These results show that both transport and regulation are specific for TA and cannot be replaced by genes present in the genome of *P. putida* KT2440.

In addition, we observed a transient PCA accumulation in the supernatant of KT2440 pBT’T_*tph* grown with TA as a sole source of carbon and energy (Fig. S4). These results indicated that the *tph* genes were functional in KT2440 and produced PCA, however further strain optimization is required to co‐regulate the upper (TA to PCA) and lower (PCA cleavage) pathways.

EG is industrially produced at large volumes and it is used in a range of applications, production of PET being one of them. Aerobic metabolism of EG was described in some *Pseudomonas* species (Mückschel *et al*., 2012; Franden *et al*., 2018; Orellana‐Saez *et al*., 2019). Functionally redundant periplasmic quinoproteins have been found to catalyse the initial conversion of EG to glycolaldehyde (Mückschel *et al*., 2012; Wehrmann *et al*., 2017). In *P. putida* KT2440 this function is assigned to PedE (PP_2674) and PedH (PP_2679) (Wehrmann *et al*., 2017). In the next step, catalysed by cytoplasmic aldehyde dehydrogenases PedI (PP_2680) and PP_0545 in KT2440, glycolaldehyde is converted to glycolate, followed by oxidation to glyoxylate by the activity of GlcDEF (Franden *et al*., 2018). All of the genes encoding the enzymes involved in the oxidation of EG to glyoxylate have been identified in the strain GO16 (Fig. 4A; Table 2).

**Table 2:** Comparison of the genes encoding enzymes involved in the sequential oxidation of ethylene glycol (EG) to glyoxylate between *P. umsongensis* GO16 and *P. putida* KT2440.

Gene	Locus	Function	Query cover (%)	Identity AA (%)	Reference gene
*pedI*	F6476_19660	Aldehyde dehydrogenase	100	92	PP_2680
*pedH*	F6476_19665	Quinoprotein ethanol dehydrogenase	99	91	PP_2679
*pedE*	F6476_19690	PQQ dependent dehydrogenase	100	94	PP_2674
*aldB‐I*	F6476_27375	Aldehyde dehydrogenase	100	92	PP_0545
*gcl*	F6476_01220	Carboxylate ligase	100	86	PP_4297
*hyi*	F6476_01215	Hydroxypyruvate isomerase	100	78	PP_4298
*glxR*	F6476_01210	Tartronate reductase	100	86	PP_4299
*ttuD*	F6476_01205	Hydroxypyruvate reductase	99	81	PP_4300
*ttuE*	F6476_01200	Pyruvate kinase	99	79	PP_4301
*glcC*	F6476_01190	Glc operon transcriptional regulator	98	73	PP_3744
*glcD*	F6476_01185	Glycolate oxidase FAD‐linked subunit	100	86	PP_3745
*glcE*	F6476_01180	Glycolate oxidase FAD‐binding subunit	100	72	PP_3746
*glcF*	F6476_01175	Glycolate oxidase Fe‐S subunit	100	77	PP_3747
*glcG*	F6476_01170	Uncharacterized protein	90	73	PP_3748
*glcB*	F6476_01165	Malate synthase	99	63	PP_0356
*glcA*	F6476_01160	Glycolate permease	100	81	PP_4735
*maeB*	F6476_01155	Malate dehydrogenase/malic enzyme	98	71	PP_5085

The growth rate, carbon depletion and biomass yield were compared when GO16 was cultivated with TA, EG or glucose (Table 3, Fig. S5). EG supplied as a sole source of carbon and energy supports the biomass formation in *P. umsongensis* GO16. In comparison with the growth with TA, EG yields biomass 0.4 g l^−1^ of cell dry weight (CDW), which is fourfold lower than CDW observed with the corresponding amount of carbon (1.96 g_C_ l^−1^) of TA (Table 3). When TA was used as a sole source of carbon and energy the specific growth rate was twofold lower compared to the specific growth rate with glucose. However, the final biomass and biomass yield were similar when GO16 was cultivated with glucose or TA as a sole carbon and energy source (Table 3).

**Table 3:** Comparison of growth characteristics of *P. umsongensis* GO16 when glucose, TA and EG were used as a sole source of carbon and energy. Three carbon sources were used in amounts to provide 1.96 g_C_ l^−1^. The specific growth rate was calculated for the exponential phase of growth

Carbon source	Final biomass (CDW; g l^−1^)	Specific growth rate (h^−1^)	Specific rate of C consumption (g l^−1^ h^−1^)	Biomass yield (g_CDW_/g_C_)
Glucose	1.30 ± 0.06	0.22 ± 0.01	0.26 ± 0.02	0.30
TA	1.60 ± 0.07	0.17 ± 0.01	0.18 ± 0.02	0.35
EG	0.40 ± 0.13	0.09 ± 0.03	0.05 ± 0.02	0.15

The catabolic pathway that allows biomass formation from EG in *P. putida* JM37 proceeds *via* Gcl pathway including a glyoxylate carboxyligase (Mückschel *et al*., 2012). While the wild type KT2440 contains the genetic capacity to form biomass from EG, it can only utilize it as an energy source (Li *et al*., 2019). It was shown that the repression of the *gcl* operon is the reason for this, and once this repression is removed, KT2440 can efficiently grow with EG as a sole source of carbon and energy (Li *et al*., 2019). In GO16 the *gcl* operon was identified (Table 2). This operon is regulated by a LysR transcriptional regulator (*ttdR*) and followed by carboxylate ligase (*gcl*), hydroxypyruvate isomerase (*hyi*), tartronate semialdehyde reductase (*glxR*), hydroxypyruvate reductase (*ttuD*) and pyruvate kinase (*ttuE*). The *gcl* operon is followed by the *glc* operon, consisting of a regulator *glcC* and genes encoding the subunits of a glycolate oxidase, *glcDEF*.

It is worth noting that during the cultivation of GO16 with EG as a sole source of carbon and energy we did not observe the formation of EG oxidation products, glycolate, glyoxylate and oxalate, which was the case when *P. putida* KT2440 was grown in the presence of EG (Li *et al*., 2019).

### PHA metabolism

PHAs are a family of biological polyesters which represented 1.2% of the global bioplastic market in 2019 (European Bioplastics, 2019). PHAs are bacterial carbon and energy storage polyesters usually accumulated as a response to stress (Rehm, 2010). They are grouped into scl‐ polymers, containing (*R*)‐3‐hydroxyalkanoic acids with four or five carbon atoms with polyhydroxybutyrate (PHB) as a typical example, and mcl‐ polymers of (*R*)‐3‐hydroxyalkanoic acids containing 6‐12 carbon atoms (Sudesh *et al*., 2000). With over 150 known PHA monomers, (*R*)‐3‐hydroxyalkanoic acids, PHAs have highly diverse material properties and therefore a broad range of applications (Rehm, 2010).

*P. umsongensis* GO16 has a typical mcl‐PHA synthesis genes organization, with a *phaC1ZC2D* cluster (Table 4). This cluster is well conserved among the mcl‐PHA producing bacteria, and the two PhaCs encoded by it belong to class II PHA synthases typically involved in the synthesis of mcl‐PHA (Chek *et al*., 2019). We have identified an additional putative PHA synthase that also belongs to class II (Table 4), potentially contributing to mcl‐PHA synthesis in GO16. This synthase is located outside of other PHA metabolism gene clusters, and it has no PHA related genes in its proximity.

**Table 4:** Comparison of the genes encoding proteins involved in the synthesis and degradation of PHA present in *P. umosongensis* GO16 and those of related organisms. Gene codes starting with PP_, P or WP_ correspond, respectively, to *P. putida* KT2440, *Pseudomonas oleovorans* or *Pseudomonas extremoaustralis*.

Gene	Locus	Function	Query cover (%)	Identity AA (%)	Reference gene
*phaC1*	F6476_32385	Poly(3‐hydroxyalkanoate) synthase 1	100	82	PP_5003
*phaZ1*	F6476_32380	Poly(3‐hydroxyalkanoate) depolymerase	99	91	PP_5004
*phaC2*	F6476_32375	Poly(3‐hydroxyalkanoate) synthase 2	100	74	PP_5005
*phaD*	F6476_32370	TetR family transcriptional regulator	99	77	PP_5006
*GA2*	F6476_32365	Granule associated protein	65	66	PP_5007
*GA1*	F6476_32360	Granule associated protein	100	61	PP_5008
*GA3*	F6476_32350	Putative granule associated protein	98	57	PP_5010
*PHA synthase*	F6476_07555	Poly(3‐hydroxyalkanoate) synthase	94	49	P26494
*phaZ2*	F6476_23055	PHB depolymerase/α/β hydrolase	88	37	P26495
*phaC3*	F6476_22230	Class I poly(3‐hydroxyalkanoate) synthase	99	73	WP_042946539
*phaA*	F6476_22225	Acetyl‐CoA acetyltransferase	100	82	WP_010563427
*phaB*	F6476_22220	Acetoacetyl‐CoA reductase	98	80	WP_010563428
*GA4*	F6476_22205	Granule associated protein	97	66	WP_003464225
*phbF*	F6476_22200	PHA synthesis repressor	98	69	WP_010563433

**Fig. 5:**
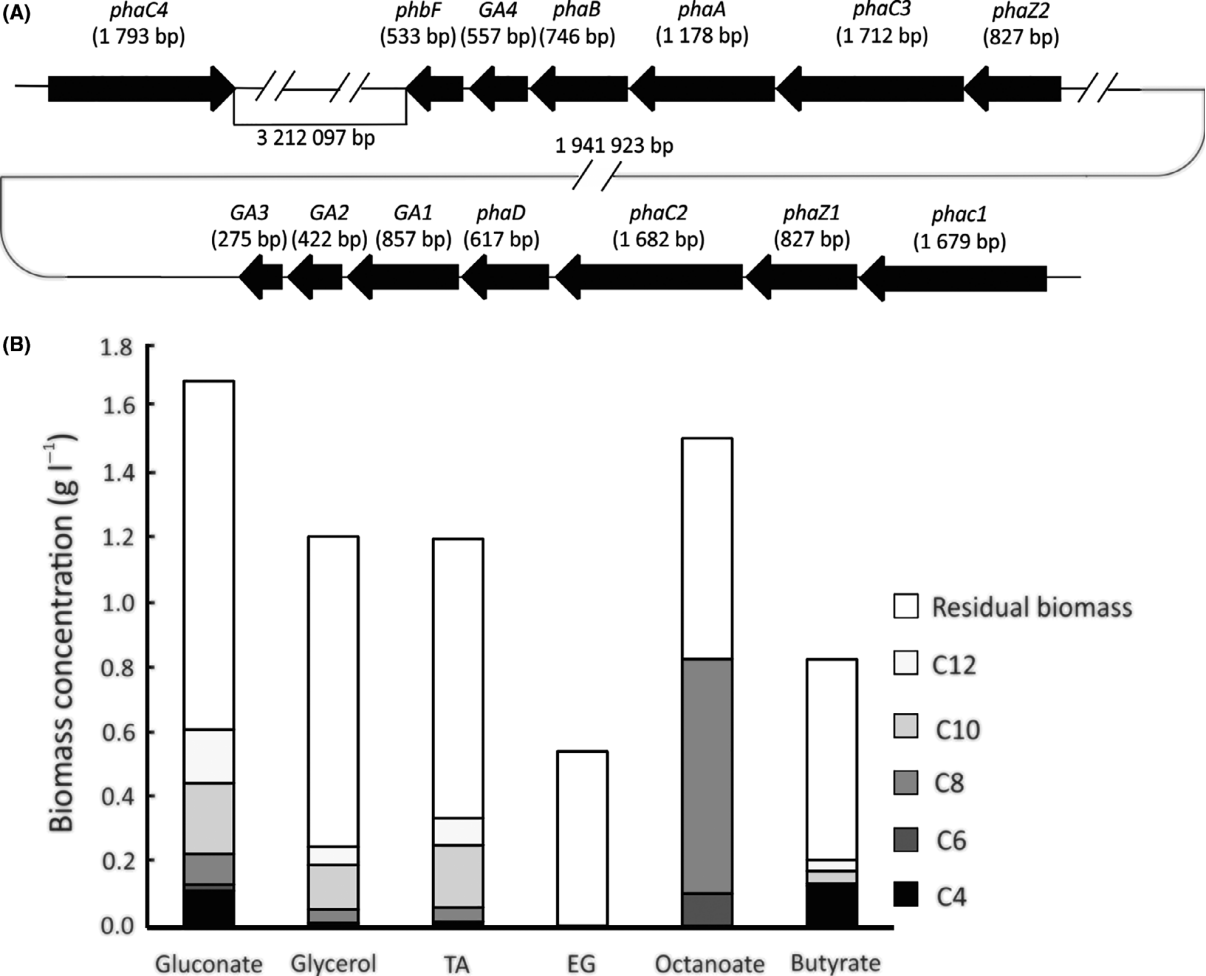
(A) Organization of the PHA metabolism genes in *P. umsongensis* GO16. (B) PHA accumulation and monomer composition (mol%) from carbon unrelated (sodium gluconate, glycerol, TA and EG) and carbon related substrates (sodium octanoate, sodium butyrate). The values represent the average of three independent biological replicates and correspond to the biomass accumulated in 48 h.

Furthermore, the *P. umsongensis* GO16 chromosome encodes a scl‐PHA synthesis pathway (Fig. 5A; Table 4). While the organization of the genes encoding a class I PHA synthase (*phaC3*), acetyl‐CoA acetyltransferase (β‐ketothiolase; *phaA*), acetoacetyl‐CoA reductase (*phaB*) is the same as in a scl‐PHA model organism *Cupriavidus necator* H16, in this model organism the *phaCAB* operon is followed by the *phaR* encoding the PHA synthesis repressor (Pohlmann *et al*., 2006). The *phaCAB* genes of GO16 are followed by two genes encoding hypothetical proteins, and genes encoding a phasin family protein and PhaR repressor (Table 4). One of the two hypothetical proteins, positioned immediately after the acetoacetyl‐CoA reductase, shows 99% identity with AraC transcriptional regulators found in *Pseudomonas* species (Gallegos *et al*., 1997). The second hypothetical protein from this cluster contains a DUF3141 domain, also found in PHA synthase of class III involved in scl‐PHA synthesis (Batista *et al*., 2016; Chek *et al*., 2019).

While mcl‐PHA accumulation is widely reported in pseudomonads, scl‐PHA production is not a common characteristic of *Pseudomonas* species (Diard *et al*., 2002). *P. oleovorans* group including *P. oleovorans*, *P. pseudoalcaligenes*, *Pseudomonas* sp., as well as recently described *P. extremoaustralis* and *Pseudomonas* sp. MPC6 are examples of *Pseudomonas* species that have the capacity to accumulate scl‐PHA when grown with sodium octanoate (Diard *et al*., 2002; Lopez *et al*., 2009; Catone *et al*., 2014; Orellana‐Saez *et al*., 2019). In *P. extremoaustralis*, the scl‐PHA, polyhydroxybutyrate (PHB) cluster *phaRBAC* is located in a genomic island of 32.3 kb containing 28 ORFs (Ayub *et al*., 2007). We have also identified an integrase 3 ORFs upstream of the scl‐PHA synthase, as well as three transposases (4806223‐4807659, 4818701‐4820131, 4837609‐4837980), suggesting that scl‐PHA gene cluster could have been acquired by horizontal gene transfer.

We investigated the ability of GO16 to form mcl‐PHA and scl‐PHA using a variety of growth substrates. GO16 accumulated mcl‐PHA when grown with gluconate, TA, glycerol, octanoic acid, but no PHA accumulation was observed when EG was used as a sole source of carbon and energy (Fig. 5B). Typically, when PHA monomer‐unrelated substrates were used, C10 was the major monomer detected with approximately 50 mol% (0.13–0.20 g l^−1^). We observed a higher fraction of C12, up to 30 mol% (0.05–0.15 g l^−1^) in mcl‐PHA accumulated by GO16 in comparison with mcl‐PHA accumulated by *P. putida* KT2440, which is also a model organism for mcl‐PHA accumulation (Sohn *et al*., 2010; Davis *et al*., 2013). In addition to typical mcl‐PHA monomers, C4 monomer was identified when butyrate (61 mol%; 0.11 g l^−1^), gluconate (18 mol%; 0.10 g l^−1^) or TA (1 mol%; 0.01 g l^−1^) were used as the substrates. When butyrate was used, C4 was predominant monomer, with C8 (3 mol%; 0.007 g l^−1^), C10 (19 mol%; 0.036 g l^−1^) and C12 (17 mol%; 0.032 g l^−1^) also present (Fig. 5B).

Besides the natural capabilities of GO16, we also tested the accumulation of PHA in KT2440 containing the pBT’T_*tph* plasmid when grown with TA as a sole source of carbon and energy. The biomass accumulated under nitrogen limitation was 0.36 g l^−1^ CDW, and 17% CDW (0.0612 g l^−1^) was PHA. The major detected monomer was C10 (75 mol%), followed by C8 (17 mol%) and C12 (8 mol%). The biomass achieved by KT2440 pBT’T_*tph* was threefold lower compared to GO16 grown under the same conditions, and total PHA amount was 4.9‐fold lower. These results show the potential to use the *tph* genes of GO16 for the upcycling of PET monomers in other bacterial species.

**Fig. 6:**
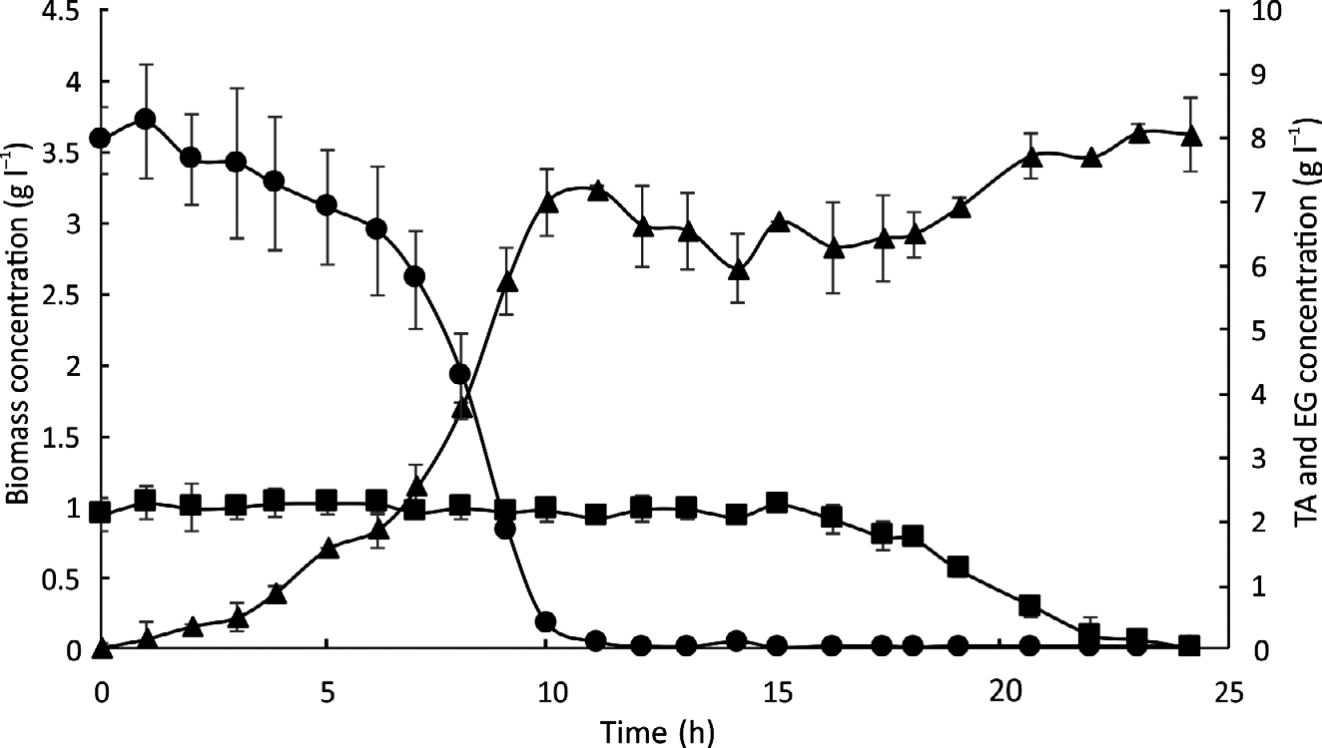
Growth and substrate consumption of *P. umsongensis* GO16 on minimal salts medium (MSM_full_) supplemented with an equimolar synthetic mixture of TA and EG to a final concentration of 40 mM (8.4 g l^−1^ TA, 2.48 g l^−1^ EG), mimicking enzymatically hydrolysed PET, in a 5 l bioreactor with a 3 l working volume at 30°C. The figure shows biomass accumulation (CDW; g l^−1^; ▲), TA utilization (g l^−1^; ●) and EG utilization (g l^−1^; ■). Error bars represent the standard deviation of three biological replicates.

We assessed the potential of GO16 for the upcycling of PET by analysing the growth and PHA production of *P. umsongensis* GO16 using equimolar mixture of TA and EG as carbon sources. Under PHA non‐accumulating conditions, TA and EG were depleted and a final CDW of 3.6 g l^−1^ was achieved, resulting in a total yield of 0.74 g_CDW_ g_C_^−1^. GO16 showed preferential utilization of TA over EG with utilization of EG beginning after complete degradation of TA (Fig. 6). After TA was completely depleted, a lag period of approximately 5 h was observed prior to commencement of EG metabolism. TA contributed 3.2 g l^−1^ CDW, with total TA utilization occurring within 10 h, resulting in a yield of 0.38 g_CDW_ g_TA_^−1^ or 0.82 g_CDW_ g_C_^−1^. The consumption of EG contributed 0.4 g l^−1^ CDW, resulting in a yield of 0.2 g_CDW_ g_EG_^−1^ or 0.42 g_CDW_ g_C_^−1^. A maximum specific growth rate (μ) of 0.37 h^−1^ was observed when utilizing TA, and a 15‐fold lower maximum specific growth rate (μ) of 0.024 h^−1^ was recorded during EG utilization compared to TA. Total depletion of EG occurred within 8 h since the start of EG consumption.

**Fig. 7:**
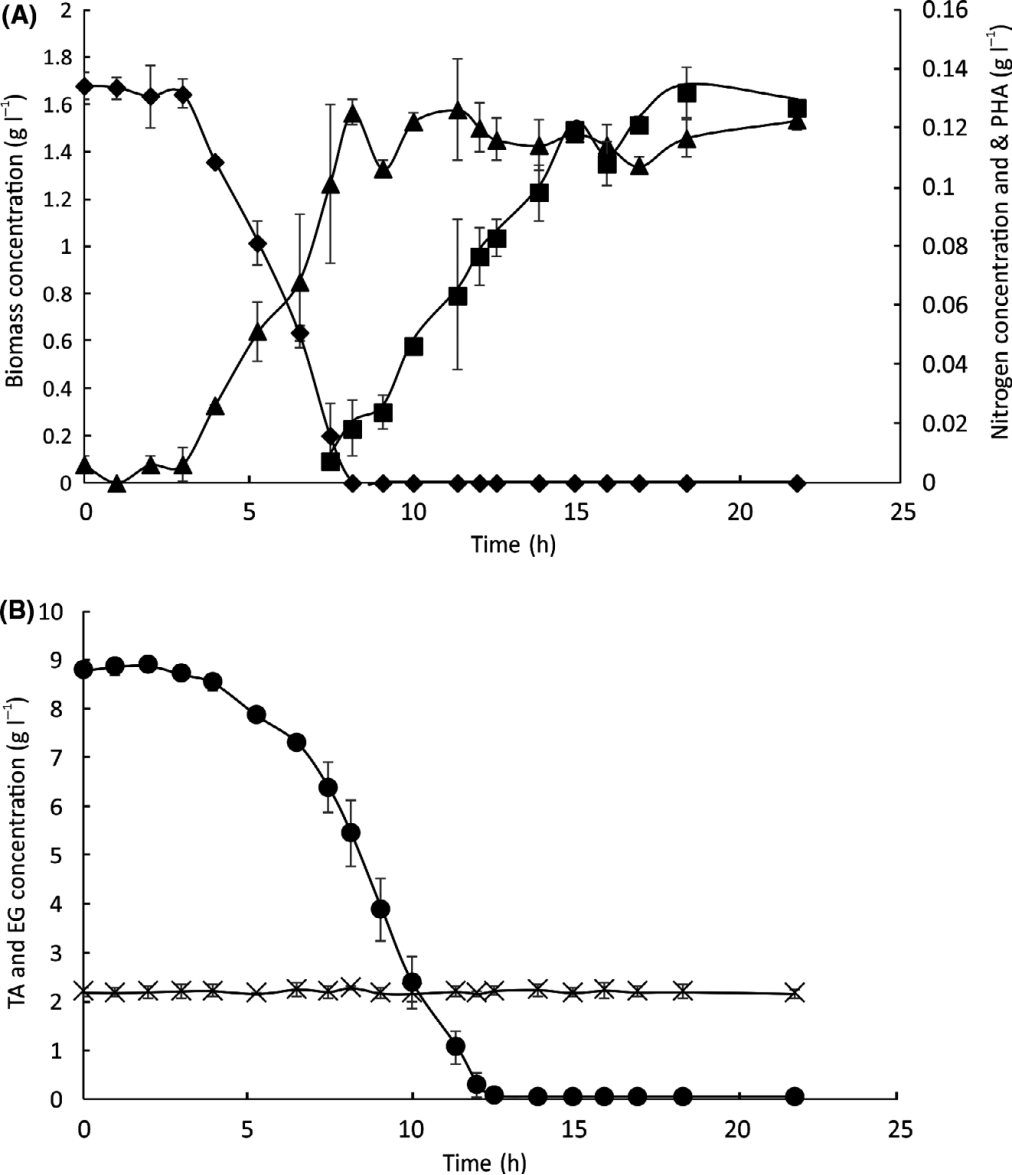
Growth and PHA accumulation of *P. umsongensis* GO16 on minimal salts media (MSM_lim_) supplemented with an equimolar synthetic mixture of TA and EG to a final concentration of 40 mM (8.4 g l^−1^ TA, 2.48 g l^−1^ EG), mimicking enzymatically hydrolysed PET, in a 5 l bioreactor with a 3 l working volume at 30°C. The figure shows (A) biomass accumulation (CDW; g l^−1^; ▲), nitrogen utilization (g l^−1^; ♦) and PHA accumulation (g l^−1^; ■) (B) TA (●) and EG (×) utilization. Error bars represent the standard deviation of three biological replicates.

Under PHA accumulating conditions, a final CDW of 1.5 g l^−1^ was achieved (Fig. 7), a twofold decrease compared to cultivation of *P. umsongensis* GO16 under non‐limiting conditions. Nitrogen was completely exhausted by 8 h, leading to the onset of PHA accumulation. A total PHA content of 0.13 g l^−1^ (9% CDW) was achieved. A consumption rate of 0.97 g (l h)^−1^ TA was observed after nitrogen depletion (Fig. 7) with complete TA utilization occurring within 12 h. No EG consumption was observed, similar to what we have observed in shake flask experiments when EG was used as a sole source of carbon and energy under PHA accumulating conditions. The medium chain length PHA produced by *Pseudomonas* sp. GO16 from the equimolar synthetic mixture of TA and EG consisted of C_10_ (53 mol%), C_8_ (25 mol%) and C_12_ (24 mol%). These results indicate that upcycling of hydrolysed PET is possible mainly at the expense of TA, which is preferentially used over EG by *P. umsongensis* GO16.

## Conclusions

In this work we have analysed the complete genome of *P. putida* GO16, a promising tool for the upcycling of PET hydrolysis monomers TA and EG (Tiso *et al*., 2020). The central metabolism of GO16 shows similarities with other species of *Pseudomonas*. For instance, glucose is most likely metabolized via the periplasmic conversion to gluconate and its complete metabolism combines the ED, EMP and PP pathways. This allows for the production of a larger surplus of reducing equivalents compared to the canonical glycolysis and it is linked to a better fitness in stressful environments including demanding bioconversions such as those required for the degradation of aromatics (Chavarria *et al*., 2013). Related to this, *P. umsongensis* GO16 is capable of degrading a wide diversity of aromatics such as naphthalene, due to the presence of pENK22, and most notably TA, which is uncommon in *Pseudomonas* species. The presence of this pathway in GO16 enables the synthesis of different molecules of interest derived from the hydrolysis of PET. These include other aromatic molecules (Kim *et al*., 2019), but also the products of the cleavage of the aromatic ring and posterior modifications, some of which containing two or more functional groups and can be used as building blocks for synthesizing different types of polymers (Johnson *et al*., 2019). One example is adipic acid which could be obtained by the recombinant expression of a protocatechuate decarboxylase transforming the protocatechuate obtained from TA into catechol (Johnson *et al*., 2016). Catechol can then be cleaved by the chromosomally encoded catechol‐1,2‐dioxygenase to render the adipic acid precursor *cis,cis*‐muconate.

Another interesting feature of *P. umsongensis* GO16 is the high abundance of genes related to the metabolism of lipids including several involved in different stages of β‐oxidation. Also related to the metabolism of lipids, GO16 harbours genes for the synthesis of both scl‐ and mcl‐PHA. C4 monomer was dominant when butyrate was used as a substrate. However, the other tested substrates drove biosynthesis of predominantly mcl‐PHA monomers. It is worth highlighting that the proportion of longer acids (e.g. C12) is enriched in GO16 compared to other *Pseudomonas* species.

As a result of the complete sequencing and genome analysis of *P. umosongensis* GO16, we have been able to identify some key properties of this organism. In particular, we have identified some aspects of its metabolism that make it a versatile workhorse for biotransformation using PET and its degradation products as feedstocks that could facilitate the development of bio‐based recycling strategies for this critical polymer with the potential for its conversion to not only one but two biodegradable polymers (scl‐PHA and mcl‐PHA).

## Experimental procedures

### Bacterial strain, medium and growth conditions

*P. umsongensis* GO16 (accession number NCIMB 41538, NCIMB Aberdeen, Scotland, UK) was inoculated from glycerol stock onto mineral salts medium (MSM) solidified with 1.5% agar supplemented with 4.4 g l^−1^ (20 mM) disodium terephthalate (TA; Sigma‐Aldrich, UK). MSM contained 9 g l^−1^ Na_2_HPO_4_·12H_2_O, 1.5 g l^−1^ KH_2_PO_4_ and 1 g l^−1^ (MSM_full_) or 0.25 g l^−1^ (MSM_lim_) NH_4_Cl. Prior to inoculation MSM was supplemented with MgSO_4_ (200 mg ml^−1^) and trace elements (per litre: 4 g ZnSO_4_∙7H_2_O; 1 g MnCl_2_∙4H_2_O; 0.2 g Na_2_B_4_O_7_∙10H_2_O; 0.3 g NiCl_2_∙6H_2_O; 1 g Na_2_MoO_4_∙2H_2_O; 1 g CuCl∙2H_2_O; 7.6 g FeSO_4_∙7H_2_O).

To test the growth of GO16 with the volatile aromatic hydrocarbons naphthalene, a single colony was cultured overnight in glucose and then diluted 1/100 when transferred into a flask with 50 ml of M9 minimal medium supplemented with vitamins and trace elements (Harwood and Cutting, 1990). Naphthalene was supplemented *via* vapour phase from an open eppendorf tube containing crystals of the substrate (10 mg) and suspended above the culture. Flasks were incubated in an orbital shaker at 30°C and 200 rpm for 4 days monitoring the absorbance at 600 nm periodically. Growth was confirmed by comparison with control flasks without substrate. For testing the growth using other soluble aromatic substrates, cultures of 200 µl of M9 supplemented with 5 mM of the aromatic molecule (all from Sigma‐Aldrich, UK) used as a carbon source, were incubated in a Clariostar microplate reader (BMG Labtech, Germany) at 30°C by measuring the optical density of the cultures at 600 nm.

For the growth and PHA accumulation analysis, a single colony was inoculated into 3 ml of MSM_Full_ supplemented with various carbon sources (sodium gluconate, TA, EG, sodium butyrate, sodium octanoate, glycerol) in the amount corresponding to 1.96 g_C_ l^−1^, and incubated for 18 h at 200 rpm and 30°C. The seed culture (1 ml) was inoculated into 250 ml Erlenmeyer flasks containing 50 ml MSM_lim_ media supplemented with the corresponding carbon source and incubated for 48 h at 200 rpm and 30°C. The cells were harvested by centrifugation at 5000 g for 10 min at 4°C (Benchtop 5430R centrifuge; Eppendorf, Germany) and washed with 10 ml of phosphate buffer (50 mM, pH 7). The pellets were frozen at −80°C.

For bioreactor experiments, the pre‐inoculum was prepared by inoculating 250 ml Erlenmeyer flasks containing 50 ml MSM_full_ with 1 ml of seed culture supplemented with 20 mM TA and 20 mM EG. Flasks were incubated in a shake incubator (New Brunswick Scientific, Innova 44; USA) for 18 h at 200 rpm and 30°C. Optical density readings (OD_540_) of the seed culture was taken prior to bioreactor inoculation using a spectrophotometer (Spectrophotometer 6300, Jenway; UK) at 540 nm.

Batch fermentation experiments were carried out in a 5 l Biostat B bioreactor (Sartorious, Germany), containing 3 l of MSM_full_ or MSM_lim_ broth supplemented with 40 mM TA and EG. The bioreactor was set up with 5% (v/v) inoculum of cells, with an OD_540_ of 3.5 ± 0.49. Air was supplied at a constant rate of 3 l min^−1^ (1 VVM) throughout the fermentation and dissolved oxygen (DO) was maintained at a minimum of 20%, via a control loop. Impeller speed was set to a minimum of 500 rpm and maximum of 1500 rpm. Temperature was maintained constant at 30°C. The pH of the culture was maintained at a value of pH 7 by the automatic addition of 20% (v/v) NH_4_OH or 15% (v/v) H_2_SO_4_. DO and rpm were monitored online by BioPAT MFCS/win fermentation data acquisition software (Sartorious; Germany). 2 ml samples were taken in duplicate at hourly intervals for CDW, carbon, nitrogen and PHA analysis.

To analyse the dynamics of growth and carbon depletion, *P. umsongensis* GO16 and *P. putida* KT2440_*tph* were grown in 50 ml MSM_full_ with glucose, TA or EG as a sole source of carbon and energy. The carbon and energy source was supplemented in an amount to provide 1.96 g_C_ l^−1^. The cultures were incubated for 48 h at 200 rpm and 30°C, and samples were withdrawn at 6 h intervals. OD was measured at absorbance 540 nm (JENWAY 6300 spectrophotometer; Cole‐Parmer, Staffordshire, UK) and 2 ml of supernatants were kept for carbon consumption analysis. The cells were harvested by centrifugation, freeze dried using a Labconco^®^ (Fisher Scientific) freeze‐dryer for CDW determination. The supernatant was retained, filtered and analysed by HPLC for carbon depletion.

For the purpose of the genomic DNA isolation, Lysogeny broth (LB; Sigma‐Aldrich, Ireland) medium was used for the cultivation of *P. umsongensis* GO16. A single colony of *P. umsongensis* GO16 was inoculated into 4 ml of LB and cultivated for 16 h at 200 rpm and 30°C. The cells were harvested by centrifugation at 16 000 *g*, 5 min, 4°C (Benchtop 5430R centrifuge; Eppendorf, Hamburg, Germany).

#### Genomic DNA extraction

The total DNA was extracted using Blood and Cell culture DNA midi kit (QIAGEN, Germany) according to manufacturer’s instructions for the lysis of bacteria. The quality and concentration of isolated DNA were verified by Qubit® Fluorometer (Thermo Fisher Scientific, Ireland) according to the manufacturer’s instructions.

#### Genome sequencing and analysis

The genome of *P. umsongensis* GO16 was sequenced by BaseClear BV (Leiden, NL). Using Illumina HiSeq platform and PacBio RSII platform a 300 bp paired‐end library and 10 kb library respectively were prepared. This was followed by a *de novo* assembly of the reads and an automated gap closure using GapFiller version 1.10.

Sequencing of *P. umsongensis* GO16 total DNA (BaseClear BV, Leiden, The Netherlands) yielded 5 contigs. Specific primers corresponding to the ends of these contigs were designed and used to amplify fragments from the total DNA using Q5^®^ High‐Fidelity DNA Polymerase (New England BioLabs, Ipswich, MA, USA). The amplified products were cloned into pGEM^®^‐T Easy vector (Promega, Madison, WI, USA) and sequenced (GATC Biotech, Ebersberg, Germany). The resulting sequences were assembled using DNASTAR^®^ Lasergene^®^ Genomics Suite Software (Thermo Fisher Scientific, Waltham, MA, USA). These additional end sequences and the 5 assembled contigs were orientated, overlapped and ordered relative to the genome of the closely related *P. umsongensis* BS3657 strain. Each sequence was mapped to the BS3657 strain using ‘blastn’, part of the Basic Local Alignment Search Tool (blast) suite of software (Altschul *et al*., 1990). The relative genomic coordinates for each sequence’s location were then extracted and used to merge all contigs into one larger chromosome. The phylogenetic analysis was conducted by an alignment‐free genome‐wide comparison of coding regions using a composite vector approach. The algorithm compared the amino acid counts of all predicted proteins and was implemented with the online tool cvtree3 (Qi *et al*., 2004; Zuo and Hao, 2015). Blast atlases were generated by gview java package software (https://server.gview.ca; Petkau *et al*., 2010) by carrying out genome‐wide tblastx searches between GO16 and each of the representative *Pseudomonas* genomes. The same approach was taken for comparisons between plasmid pENK22 and other plasmids containing naphthalene‐catabolic genes. Regions on each genome reporting a BLAST hit above the threshold cut‐off (80% identity, minimum HSP length of 100 bp, and expect value of 1e−10) were considered a valid match and represented in the figures. Genome alignments were generated using Progressive Mauve (Darling *et al*., 2004). BLAST searches were performed at the NCBI suite (https://blast.ncbi.nlm.nih.gov/Blast.cgi; Altschul *et al*., 1990) with the ‘blastp’ algorithm using either specific databases (e.g. *P. putida* KT2440) or the Swissprot database when running untargeted searches. BLAST searches against the genome of *P. umsongensis* GO16 were carried out locally with the Genome Workbench suite of NCBI (https://www.ncbi.nlm.nih.gov/tools/gbench/).

#### Generation of *P. putida* KT2440 pBT’T_*tph*, pBT’T_*tphA2A3BA1K* and pBT’T_*tphRA2A3BA1*

Based on the available genome sequence of *P. umsongensis* GO16, the *tph* operon (1 982 772‐ 1 988 856 in CP044409.1; 6128 bp) was synthesized (TwistBioscience, UK) and cloned into pBT’T (Koopman *et al*., 2010) vector using NEBuilder^®^ HiFi DNA Assembly Master Mix (NEB, UK). To allow Gibson assembly of the insert and the vector, the *tph* operon was amplified using the following primers RF 5′‐CAG TGA GCC CCA TCC CAA CAT CAA AGC ATA‐3′ and GR 5′‐CCG ACG TCG CAT GCT CCT CTA GA‐3′, while pBT’T was amplified using V1F 5′‐TCT AGA GGA GCA TGC GAC GTC GG‐3′ and V1rR 5′‐TAT GCT TTG ATG TTG GGA TGG GGC TCA CTG‐3′. 100 ng of vector and twofold molar excess of insert (351.4 ng of insert RT) were incubated with NEBuilder^®^ HiFi DNA Assembly Master Mix as recommended by the manufacturer. Gibson reaction (10 µl) was incubated in ice 1 h and transferred by heat shock into *E. coli* DH5α chemically competent cells. Prior to plating on selective media, cells were allowed to recover at 37°C for 2 h. The pBT’T_*tph* construct was verified by sequencing (Eurofins, Ireland) transformed into *P. putida* KT2440 (Choi *et al*., 2006) and the positive transformants were selected on MSM_full_ with TA as the sole carbon and energy source and confirmed by colony PCR using the primers DHD_FWD 5′‐CTA TCG CGC AGC CAT GGA TCT ATG A‐3′ and DHD_REV 5′‐TGG ACC TTG GTG GTA ATG ACC TTG CG‐3′, which amplify the region of the *tph* operon between *tphA2* and *tphA1* giving a product of 1809 bp. The stability of the plasmid after growth on TA was verified by miniprep (Qiagen, UK) followed by digestion with SphI, and PCR of the backbone and the *tph* genes with primers V1F and V1rR and RT 5′‐CAG TGA GCC CCA TCC CAA CAT CAA AGC ATA‐3′ and GR respectively (see Fig. S6 for details). The growth was tested in 50 ml MSM_lim_ as described above. The wild type *P. putida* KT2440 was used as a negative control, as this strain does not grow with TA as a sole source of carbon and energy.

The *tphA2A3BA1K* fragment (5160 bp) was synthesized (TwistBioscience, UK) and cloned into pBT’T (Koopman *et al*., 2010) vector using NEBuilder^®^ HiFi DNA Assembly Master Mix (NEB, UK). The insert was amplified using the following primers A2‐K F 5′‐CAG GAG GTC AAC AAT GAA CAT CAT TAC TGA‐3′ and A2‐K R 5′‐ACG TCG CAT GCT CCT CTA GAT TAA AGC GTG‐3′, while pBT’T was amplified using V2F 5′‐CAC GCT TTA ATC TAG AGG AGC ATG CGA CGT‐3′ and V2R 5′‐TCA GTA ATG ATG TTC ATT GTT GAC CTC CTG‐3′. The ligation and transformation were performed as described above.

The insert *pcaRtphA2A3B1A1* (4715 bp) that includes the native regulator, but excludes the transporter was synthesized by TwistBioscience (UK) and cloned into pBT’T (Koopman *et al*., 2010) vector using NEBuilder^®^ HiFi DNA Assembly Master Mix (NEB, UK). The primers used for Gibson assembly were RF 5′‐CAG TGA GCC CCA TCC CAA CAT CAA AGC ATA‐3′ and A2R 5′‐GCA TGC TCC TCT AGA CTA TGA AGG CGG CAG‐3′ for the insert, and V3F 5′‐CTG CCG CCT TCA TAG TCT AGA GGA GCA TGC‐3′ and V1rR 5′‐TAT GCT TTG ATG TTG GGA TGG GGC TCA CTG‐3′ for the vector. The ligation and transformation were performed as described above.

**Nutrient analysis**

For the analysis of TA consumption, the supernatant collected during cultivation had to be diluted so that the concentration of TA in the final preparation did not exceed 0.63 g l^−1^. A 1100series HPLC (Agilent, USA) equipped with a C18 ODS Hypersil column (125 × 3 mm, particle size 5 μm; Thermo Scientific, USA) was used, and samples were isocratically eluted using 0.4% formic acid at a flow rate of 1 ml min^−1^ and read on a UV‐vis detector at 230 nm. The TA retention time under the above conditions was 4.2 min. The PCA retention time under the above conditions was 3.2 min.

EG depletion was monitored using an Aminex HPX‐87H ion exclusion column (300 mm × 7.8 mm, particle size 9 μm; Bio‐rad). The column was maintained at 40°C and samples were isocratically eluted using 0.014 N H_2_SO_4_ at a flow rate of 0.55 ml min^−1^ and read on a refractive index detector (RID). The EG retention time under the above conditions was 23 min.

Glucose consumption was determined from supernatant by HPLC (i‐series, Shimadzu, Kyoto, Japan) using an Aminex HPX‐87H Column (300 × 7.8 mm, particle size 9 µm particle size; Bio‐rad, UK). The samples were eluted with 0.014N H_2_SO_4_ at a flow rate of 0.55 ml min^−1^.

#### PHA extraction and content determination

The polymer content was assayed by subjecting the lyophilized cells to acidic methanolysis as previously described (Lageveen *et al*., 1988). The PHA monomers’ methylesters were assayed by GC using a Hewlett‐Packard 6890N chromatograph equipped with a HP‐Innowax capillary column (30 m × 0.25 mm, 0.50μm film thickness; Agilent Technologies) and a flame ionization detector (FID), using the temperature programme previously described (Lageveen *et al*., 1988). Total PHA content was determined as a percentage of CDW.

## Conflict of interests

Author Shane Kenny is employed by the company Bioplastech. The remaining authors declare that the research was conducted in the absence of any commercial or financial relationships that could be construed as a potential conflict of interest.

## Author contributions

TN, SK, KO and JJ designed the study and supervised the research. TN, NB, SK, JU, UA and MS conducted experimental work. TN, MS, GH, HW and JJ performed the bioinformatic analyses. All authors contributed to writing the manuscript.

## Supporting information


**Table S1.** Blast atlas of *P. umsongensis* GO16 chromosomal genes. The table shows the results of the tblastx analysis comparing the annotated features present in the chromosome of *P. umsongenis* GO16 against the genomes of representative *Pseudomonas* species (see the experimental procedures section for details on the computational analysis).

**Table S2.** Blast atlas of the plasmid pENK22. The table shows the results of the tblastx analysis comparing the annotated features present in the plasmid pENK22 against representative similar plasmids (see the experimental procedures section for details on the computational analysis).

**Table S3.** Blast comparison of P. umsongensis GO16 putative genes belonging to the central metabolism. The analysis was conducted using the tblastx feature of the blast suite at NCBI (see the experimental procedures section for details on the computational analysis).

**Table S4.** Blast comparison of P. umsongensis GO16 putitave genes taking part in the metabolism of aromatics. The analysis was conducted using the blastp feature of the blast suite and the genome workbench tool at NCBI using known enzymes for comparison (see the experimental procedures section for details).

**Fig. S1.** Phylogenetic analysis of different members of the *Pseudomonas* genus. Genome‐wide comparison between *P. umsongensis* GO16 and notable *Pseudomonas* species was conducted using a composite vector approach (see the experimental procedures section in the main text for details).

**Fig. S2.** Growth profile of *P. umsongensis* GO16 in different soluble aromatics as the sole carbon source. In panels A to C cells were cultured in the microplate reader using the indicated aromatic substrates at a final concentration of 5 mM. Panel D shows bacterial growth in flasks in the presence of naphthalene vapours as the sole carbon source (see methods for details). Results correspond to the mean and standard deviation of three biological replicates.
**Fig. S3.** Growth profile and substrate consumption of *P. putida* KT2440 expressing the *tph* genes from *P. umsongensis* GO16. P. putida KT2440 was transformed with the plasmid pBT’T_tph (KT_tph) or the empty control pBT’T (WT in the plot) for comparison. Only KT_tph was able to use TA as the sole carbon source for growth (shown in red; solid line for growth and dashed line for substrate consumption). Expression of the tph genes (green lines) did not affect growth in glucose compare to the control (blue lines). Results correspond to the mean and standard deviation of three biological replicates.
**Fig. S4.** Terephthalic acid (TA) depletion and protocatechuate (PCA) accumulation in the supernatant of *P. putida* KT_*tph* grown with TA as a sole source of carbon and energy. (A) Kinetics of PCA accumulation and TA consumption when the strain was cultivated in MSM medium without nitrogen limitation (full nitrogen), or under polyhydroxyalkanoate (PHA) accumulating conditions (limited nitrogen) determined by HPLC‐UV. (B) After 12 hours of incubation in TA and N limited conditions the culture exhibits a characteristic purple colour corresponding to PCA accumulation in the supernatant (left flask). The culture with full nitrogen that does not accumulate PCA is shown for comparison (right flask). (C) Chromatograms of TA and PCA determination. The upper panel represents a supernatant after 12 hours of culturing in N limited conditions. Mid and lower panels represent, respectively, a standard of 0.075 g L‐1 of PCA and a standard with a mixture of 0.08 g L‐1 of PCA and 0.11 g L‐1 of TA.
**Fig. S5.** CDW (blue lines) and substrate consumption (red lines) of *P. umsongensis* GO16 growing on TA (upper), EG (mid) and glucose (lower panel). All cultures contained 1.96 gC L‐1. Plots show the mean and standard deviation of three biological replicates.
**Fig. S6.** Analysis of the stability of the plasmid pBT’T_*tph* in *P. putida* KT2440. The plasmid was purified from KT2440 grown on TA as the sole carbon source using a standard miniprep protocol. The plasmid preparation was digested with SphI (lane sphI) rendering the expected fragments of 6.3 and 3.8 kb. The plasmid was also used as a template for PCR reactions with oligonucleotides V1F and V1rR annealing on the backbone and producing a 3.9 kb DNA fragment (lane pBT’T), and with oligonucleotides RT and GR that render a 6.1 kb DNA product (lane RT). Size in bp of the molecular weight markers is shown for comparison.
