# Supplementary material for: Genome analysis of the metabolically versatile Pseudomonas umsongensis GO16: the genetic basis for PET monomer upcycling into polyhydroxyalkanoates
Source: Microb Biotechnol. 2021 Jan 6;14(6):2463–80. doi: 10.1111/1751-7915.13712 (PMC8601165; doi:10.1111/1751-7915.13712)
Supplement: Supplementary file 6 — Fig. S2. Growth profile of P. umsongensis GO16 in different soluble aromatics as the sole carbon source. In panels A to C cells were cultured in the microplate reader using the indicated aromatic substrates at a final concentration of 5 mM. Panel D shows bacterial growth in flasks in the presence of naphthalene vapours as the sole carbon source (see methods for details). Results correspond to the mean and standard deviation of three biological replicates. Fig. S3. Growth profile and substrate consumption of P. putida KT2440 expressing the tph genes from P. umsongensis GO16. P. putida KT2440 was transformed with the plasmid pBT’T_tph (KT_tph) or the empty control pBT’T (WT in the plot) for comparison. Only KT_tph was able to use TA as the sole carbon source for growth (shown in red; solid line for growth and dashed line for substrate consumption). Expression of the tph genes (green lines) did not affect growth in glucose compare to the control (blue lines). Results correspond to the mean and standard deviation of three biological replicates. Fig. S4. Terephthalic acid (TA) depletion and protocatechuate (PCA) accumulation in the supernatant of P. putida KT_tph grown with TA as a sole source of carbon and energy. (A) Kinetics of PCA accumulation and TA consumption when the strain was cultivated in MSM medium without nitrogen limitation (full nitrogen), or under polyhydroxyalkanoate (PHA) accumulating conditions (limited nitrogen) determined by HPLC‐UV. (B) After 12 hours of incubation in TA and N limited conditions the culture exhibits a characteristic purple colour corresponding to PCA accumulation in the supernatant (left flask). The culture with full nitrogen that does not accumulate PCA is shown for comparison (right flask). (C) Chromatograms of TA and PCA determination. The upper panel represents a supernatant after 12 hours of culturing in N limited conditions. Mid and lower panels represent, respectively, a standard of 0.075 g L‐1 of PCA and a standard with a mi [file MBT2-14-2463-s003.docx]

**Supplementary figures for ‘Genome analysis of the metabolically versatile Pseudomonas umsongensis GO16: The genetic basis for PET monomer upcycling into polyhydroxyalkanoates’**

**Figure S1. Phylogenetic analysis of different members of the *Pseudomonas* genus.** Genome-wide comparison between *P. umsongensis* GO16 and notable *Pseudomonas* species was conducted using a composite vector approach (see the experimental procedures section in the main text for details).

**Figure S2. Growth profile of *P. umsongensis* GO16 in different soluble aromatics as the sole carbon source.** In panels A to C cells were cultured in the microplate reader using the indicated aromatic substrates at a final concentration of 5 mM. Panel D shows bacterial growth in flasks in the presence of naphthalene vapours as the sole carbon source (see methods for details). Results correspond to the mean and standard deviation of three biological replicates.

**Fig. S3. Growth profile and substrate consumption of *P. putida* KT2440 expressing the *tph* genes from *P. umsongensis* GO16.** *P. putida* KT2440 was transformed with the plasmid pBT’T_*tph* (KT_tph) or the empty control pBT’T (WT in the plot) for comparison. Only KT_tph was able to use TA as the sole carbon source for growth (shown in red; solid line for growth and dashed line for substrate consumption). Expression of the *tph* genes (green lines) did not affect growth in glucose compare to the control (blue lines). Results correspond to the mean and standard deviation of three biological replicates.

**Fig. S4. Terephthalic acid (TA) depletion and protocatechuate (PCA) accumulation in the supernatant of *P. putida* KT_*tph* grown with TA as a sole source of carbon and energy.** (A) Kinetics of PCA accumulation and TA consumption when the strain was cultivated in MSM medium without nitrogen limitation (full nitrogen), or under polyhydroxyalkanoate (PHA) accumulating conditions (limited nitrogen) determined by HPLC-UV. (B) After 12 hours of incubation in TA and N limited conditions the culture exhibits a characteristic purple colour corresponding to PCA accumulation in the supernatant (left flask). The culture with full nitrogen that does not accumulate PCA is shown for comparison (right flask). (C) Chromatograms of TA and PCA determination. The upper panel represents a supernatant after 12 hours of culturing in N limited conditions. Mid and lower panels represent, respectively, a standard of 0.075 g L^-1^ of PCA and a standard with a mixture of 0.08 g L^-1^ of PCA and 0.11 g L^-1^ of TA.

**Fig. S5. CDW** (blue lines) **and substrate consumption** (red lines) **of *P. umsongensis* GO16 growing on TA** (upper), **EG** (mid) **and glucose** (lower panel)**.** All cultures contained 1.96 g_C_ L^-1^**.** Plots show the mean and standard deviation of three biological replicates.


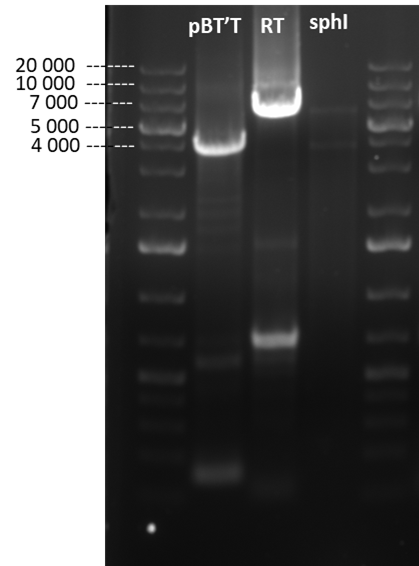


**Fig. S6. Analysis of the stability of the plasmid pBT’T_*tph* in *P. putida* KT2440.** The plasmid was purified from KT2440 grown on TA as the sole carbon source using a standard miniprep protocol. The plasmid preparation was digested with SphI (lane sphI) rendering the expected fragments of 6.3 and 3.8 kb. The plasmid was also used as a template for PCR reactions with oligonucleotides V1F and V1rR annealing on the backbone and producing a 3.9 kb DNA fragment (lane pBT’T), and with oligonucleotides RT and GR that render a 6.1 kb DNA product (lane RT). Size in bp of the molecular weight markers is shown for comparison.

**Legends for supplementary tables**

**Table S1: Blast atlas of *P, umsongensis* GO16 chromosomal genes.** The table shows the results of the tblastx analysis comparing the annotated features present in the chromosome of *P. umsongenis* GO16 against the genomes of representative *Pseudomonas* species (see the experimental procedures section for details on the computational analysis).

**Table S2: Blast atlas of the plasmid pENK22.** The table shows the results of the tblastx analysis comparing the annotated features present in the plasmid pENK22 against representative similar plasmids (see the experimental procedures section for details on the computational analysis).

**Table S3: Blast comparison of *P. umsongensis* GO16 putative genes belonging to the central metabolism.** The analysis was conducted using the tblastx feature of the blast suite at NCBI (see the experimental procedures section for details on the computational analysis).

**Table S4: Blast comparison of *P. umsongensis* GO16 putitave genes taking part in the metabolism of aromatics.** The analysis was conducted using the blastp feature of the blast suite and the genome workbench tool at NCBI using known enzymes for comparison (see the experimental procedures section for details).
